# Got herpes? (Yep, you probably do!)

**DOI:** 10.1371/journal.ppat.1006652

**Published:** 2017-11-09

**Authors:** Eain A. Murphy

**Affiliations:** 1 Department of Translational Medicine, Baruch S. Blumberg Institute of Living Science, Doylestown, Pennsylvania, United States of America; 2 FORGE Life Science, LLC, Pennsylvania Biotechnology Center, Doylestown, Pennsylvania, United States of America; The Fox Chase Cancer Center, UNITED STATES

Most scientists have a 2- to 3-minute prepared spiel about his or her research that can be given at a moment’s notice, upon request. We call this the “Elevator Speech” (or “Lift Speech” if you are British), and it is often delivered after you tell someone that you are a research scientist. While I’m prepared to explain my job, I am never prepared for the looks on individuals’ faces when I tell them, “I work on herpes,” which could be equated with the looks I might get if I asked them to borrow their toothbrush. I explain how pervasive herpesviruses are in the human population and how, on average, humans carry multiple herpesvirus infections with them for life. With looks of horror, the vehement denials begin. But this is when I remind them that chicken pox is caused by varicella-zoster virus, roseola is caused by human herpesvirus-6, that the college bout of mononucleosis they may have had was most likely caused by Epstein-Barr virus, each of which is a herpesvirus. Then I hit them with the bombshell: I tell them that I work on a virus they most likely have never heard of yet are most likely carrying in their bodies now and that, moreover, it is a significant pathogen and the cause of substantial medical burden. I work on human cytomegalovirus (HCMV), the largest human herpesvirus, with a genome size of a quarter million nucleotides encoding many viral proteins, of which we only understand the function of about two-thirds. While links have been made to certain brain tumors and atherosclerosis, if one has a healthy immune system, HCMV infections are usually inapparent. However, HCMV is highly problematic in individuals with undeveloped or compromised immune systems. With all the notoriety recently given to birth defects caused by Zika infections, these defects pale in comparison to HCMV. About 1% of United States births involved HCMV-infected babies, and 10% of those infants infected will show symptoms, making it the leading cause of congenital birth defects, including vision and hearing loss, learning disabilities, and microcephaly. Equally problematic are patients with weakened immune systems, including bone marrow transplant patients and solid organ recipients. Most individuals acquire HCMV as a toddler, after which it remains quiet and repressed by a person’s immune system, yet awaiting a chance to reawaken. In organ transplant patients, HCMV comes back with a vengeance and causes significant disease and organ rejection. HCMV is like that despicable kid in grammar school who you never really noticed before but who was the first one to kick you when you were down.

I am often asked how I came to study HCMV. As a child, I never had intentions of pursuing a scientific career. Having grown up in the Bronx with my New York City detective father and Irish immigrant mother, I assumed that I would take the normal occupational path that most of my friends pursued—either become a criminal or join the New York Police Department (NYPD) or Fire Department New York (FDNY). My father explained to me that I would do far better in life if I went to college. His persuasiveness (and the threat of filicide) convinced me to apply. I opted for those on a national “Top 10” ranking of small liberal arts colleges and decided to go to the one with the highest ranking that accepted me. So, I arrived on campus at Grinnell College in Iowa, sight unseen, with the intent of just completing 4 years, then going back to take the NYC civil servant exam. It was there that a professor, Dr. Charles Sullivan, sold me on the beauty of biology and the analytical nature of the science. I am very mechanical by nature: I fix old wristwatches and build old BMW motorcycles for recreation. So perhaps it was natural that I was fascinated by biological research because I viewed it as dissecting a complex machine and figuring out how each part interacts with another. These characteristics are completely summed up in the field of virology. There is a Spanish term commonly used in Cuba called “rascuache,” used to describe individuals who can repair items with limited resources and those who can repurpose items to accomplish a different function. To me, viruses are the “Masters of Rascuache,” and I was drawn to study them. Of course, the path wasn’t direct by any means. In between my newly found deep respect for viruses and my serious pursuit of academic endeavors, there were countless speed bumps and diversions. I pursued many things following college as I was finding myself. I hitchhiked to and through Alaska with my best friend from the Bronx, worked as an NYC union carpenter walking girders and building skyscrapers, bartended, worked in parts/repair in a Texas Harley Davidson dealership, and played countless rugby games.

However, the desire to study viruses never left me. I enrolled in a master’s program at Boston University to increase my admissibility into PhD programs. This proved to be the right move when I was admitted to the University of Iowa graduate school. There, I decided to join the laboratory of a very skilled virologist working on HCMV, Dr. Mark Stinski. Mark taught me that science should be fun, and if you don’t love what you are doing, then you are probably doing the wrong thing. After graduating, I left Mark’s lab to do a brief postdoc at Columbia University, and from there, I went to Princeton University to join the lab of Dr. Thomas Shenk. Tom’s lab was heaven to me. Beyond Tom being a very gracious mentor who truly helped shape the careers of each person in his lab, there were about 15 to 20 other highly skilled postdocs who were incredibly smart, always helpful, and quick to become lifelong friends. It was in this environment that my virology career truly blossomed. From Princeton, I became an assistant staff and then associate staff member in molecular genetics at the Cleveland Clinic, where my laboratory focused on the factors controlling HCMV latency. Following my promotion, I moved my laboratory to the Hepatitis B Foundation, where I run my academic laboratory, as well as serve as Head of Biology for a company founded by Tom Shenk making novel compounds to block viral infections.

The major focus of my laboratory is to figure out how to keep HCMV “in check.” Everyone who is infected with HCMV (about 50% of the general population by age 30 and increasing with age to 90% of 80-year-olds) has the virus for life because HCMV can hide in stem cells within your bone marrow. An initial infection is resolved by one’s immune system, which continues to keep the virus in a repressed state. However, when one’s immune system becomes weakened, it can no longer effectively block the virus from waking back up, something we call reactivation. If one could block reactivation, one could effectively block the diseases caused by HCMV. My laboratory is investigating what factors and conditions are required for the virus to reactivate. We have found specific factors that keep the virus in a repressed state as well as factors that allow for efficient reactivation of the virus. We are now working on how to manipulate these conditions to create an environment where HCMV cannot reactivate. We are not alone: there are several “competing” laboratories studying similar aspects of HCMV latency. I put competing in quotes because, in all fairness, these laboratories are run by very skilled scientists who I highly respect and who are incredibly supportive of each other. When you have a common enemy—the virus we are trying to cripple—you find that individuals with shared goals easily become some of your best friends. My friends and I are doing our best to make sure HCMV can’t kick you when you are down.

**Image 1:**
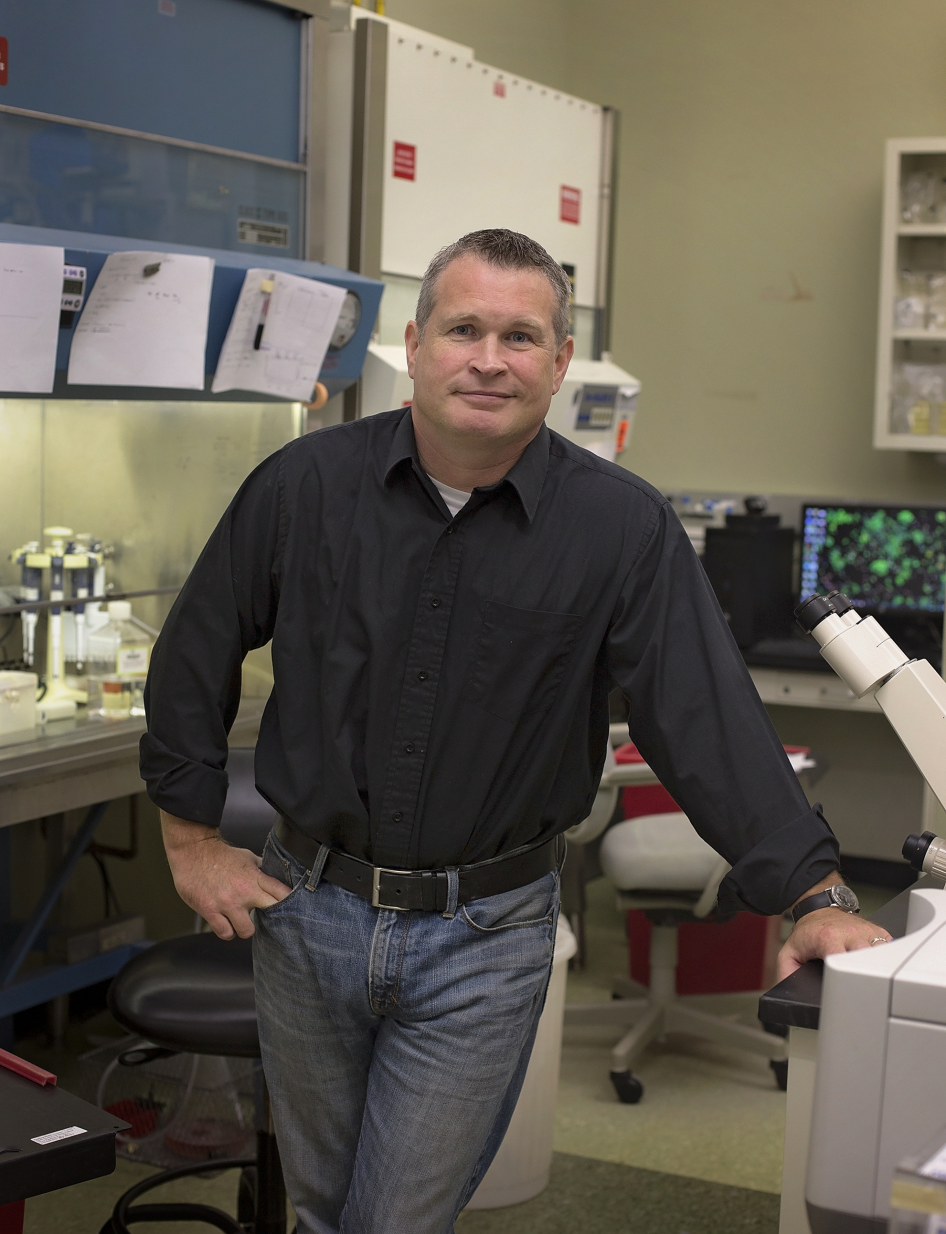
Eain A. Murphy in his laboratory where he studies herpesvirus infections.

